# High-precision SAW bandpass filtering at 1747.5 MHz for LTE applications using wavelet transform techniques

**DOI:** 10.1038/s41598-025-27145-z

**Published:** 2025-11-24

**Authors:** Hagar A. Ali, Mohamed I. Ibrahem, Hala M. Abdelkader, M. M. Elsherbini

**Affiliations:** 1 Department of Communications and Electronics Engineering , October High Institute for Engineering and Technology , 12573 Giza, Egypt; 2 Department of Electrical Engineering Shoubra Faculty of Engineering , Benha University , Cairo, Egypt

**Keywords:** WTP, SAW, IL, ECC, LTE, IDTs, Engineering, Physics

## Abstract

Recent developments in mobile communication technologies have intensified the demand for higher data rates, necessitating the use of elevated carrier frequencies and compact, high-performance, cost-effective radio frequency (RF) filters. Surface acoustic wave (SAW) filters offer notable advantages in mobile phone applications due to their low insertion loss, compact footprint, and scalable manufacturing. These attributes make them particularly well-suited to meet the growing need for affordable filtering solutions at increasingly higher operating frequencies. This paper presents the design and simulation of a wavelet transform-based surface acoustic wave (SAW) bandpass filter centered at 1747.5 MHz, optimized for GSM/LTE applications. The filter employs a multi-stage configuration enhanced by window functions such as Gaussian, Kaiser, Hanning, and Hamming to achieve precise null bandwidth control and effective side-lobe suppression. Wavelet transform integration streamlines spectral decomposition, enabling efficient frequency-domain analysis and reducing computational complexity. Finite element modeling is performed using COMSOL Multiphysics to simulate the electromechanical behavior of piezoelectric substrates, specifically quartz types. MATLAB is utilized for wavelet-domain signal processing and graphical analysis. Simulation results reveal a passband width of approximately 17.736 MHz, side-lobe attenuation below 140 dB, and stable center frequency alignment across substrate variations. FFT plots confirm strong frequency selectivity, while displacement profiles illustrate substrate-dependent acoustic wave propagation and energy confinement. The proposed wavelet-integrated SAW filter demonstrates high spectral resolution, robust frequency stability, and low-loss transmission, validating its potential for next-generation RF front-end systems.

## Introduction

Surface acoustic wave (SAW) devices have emerged as critical components in modern RF front-end systems, particularly for bandpass filtering in wireless communication^1,2^. Their compact form factor, high selectivity, and compatibility with piezoelectric substrates make them ideal for frequency-domain signal processing. Surface acoustic wave (SAW) devices benefit from inherently low wave velocities, enabling compact geometries, and exhibit minimal propagation loss, which facilitates the realization of high-quality factors (Q) in acoustic filtering applications. A key advantage of these devices lies in the straightforward fabrication of interdigital transducers (IDTs), which supports scalable and cost-effective production. This simplicity allows SAW filters to be effectively implemented across VHF and UHF frequency ranges. For instance, an IDT with 1-µm electrode spacing can yield an operating frequency near 1 GHz, contingent on the acoustic velocity of the chosen substrate material.

To address these limitations, this study introduces a novel SAW bandpass filter architecture centered at 1747.5 MHz that integrates wavelet transform principles into both the design and analysis phases. Unlike traditional Fourier-based approaches, wavelet transforms offer localized time-frequency resolution, enabling more efficient decomposition of transient signals and improved handling of non-stationary components^3^. This integration facilitates enhanced control over filter characteristics such as passband sharpness, side-lobe attenuation, and spectral confinement. To realize a filter using a surface acoustic wave (SAW) device, two precisely engineered interdigital transducers (IDTs), an input IDT and an output IDT, are fabricated on a piezoelectric substrate. These IDTs can be designed either uniformly (unapodized) or with nonuniform overlapping patterns (apodized)^3,4^. A wide range of theoretical approaches has been developed for SAW device implementation, beginning with early equivalent circuit models (ECMs)^5,6^, delta-function models^1^, impulse response models^2^, Green’s function models^7,8^, P-matrix models^9^, and coupling-of-modes (COM) models^10^. These theoretical models have undergone continuous refinement, contributing to the enhanced accuracy and versatility of SAW device analysis and design. The filter design is formulated using a combination of the impulse response model (IRM) and Mason’s equivalent circuit model (ECM) to simplify the overall design process. The designed filter is based on the multi-stage architecture of the WT/SAW filter. At each stage, the processed input signal is converted into an output that serves as the input for the following stage. This configuration enables a quantitative enhancement of filter performance by suppressing side-lobe power and strengthening the response of the main lobe. Studying different window functions to refine frequency selectivity and suppress undesired spectral leakage^4^. Finite element modelling (FEM) is conducted using COMSOL Multiphysics to simulate acoustic wave propagation and electromechanical coupling behavior, and graphical interpretation of displacement profiles^10^. MATLAB is employed for wavelet-domain signal processing, frequency-domain analysis, and the computation of susceptance and conductance parameters^11,12^.

The design parameters were chosen to align with a commercially available configuration suitable for an uplink LTE bandpass filter, and implemented based wavelet-enhanced SAW filter design that not only improves spectral resolution and computational efficiency but also enables precise tuning of key performance metrics such as insertion loss, null bandwidth, and center frequency stability. The results validate the proposed approach as a viable solution for high-performance RF filtering in next-generation communication systems.

This paper is organized as follows: Section II details the design parameters and methodology, focusing on the primary model in the frequency domain. Section III presents a comparative evaluation and explores methods to improve the response characteristics. Section IV provides an in-depth time-domain analysis conducted using COMSOL. Finally, Sections V and VI offer the conclusion and the list of references, respectively.

## Design and methodology

The simulation process is conducted in two phases: a single-stage filter and a multi-stage filter. A comparative analysis is performed between the two configurations. The simulation workflow is further divided into input parameter computation and output parameter evaluation.

### Input IDTs computation

The geometric configuration of the interdigital transducer (IDT) is a distinctive aspect of SAW filter design, representing a spatially sampled version of the IDT’s impulse response. Consequently, implementing a wavelet transform processor-based SAW device necessitates that the input IDT be designed to match the envelope of the Morlet wavelet function^3^. The mathematical formulation of the Morlet wavelet’s impulse response is presented in references^13–15^ as follows

1 $${\psi\:}_{\:}\left(t\right)=\frac{1}{\sqrt{s}}{e}^{-\frac{1}{2}{\left(\frac{t}{s}\right)}^{2}}{e}^{j{\omega\:}_{0}t}$$

Where, $\:{\omega\:}_{0}$ is the central frequency and s is the wavelet scale, let

2 $$Y\left(\omega\:\right)=\frac{\psi\:\left(\omega\:\right)\:}{\frac{\sqrt{\pi\:}}{\sqrt{s}}}={e}^{-\frac{1}{2}{s}^{2}{(\omega\:-{\omega\:}_{0})}^{2}}$$

According to^3^, the 3 dB bandwidth in megahertz (MHz) is given by

3 $${\varDelta\:f}_{-3dB}=\frac{1}{2\pi\:s}\sqrt{-2{ln}\left({10}^{\frac{-3}{20}}\right)}=\frac{0.1323}{s}$$

The number of interdigital transducers in a uniform SAW device is linked to the null bandwidth and center frequency through Eq. (4), as detailed in^5^

4 $$N = \left\lceil {\frac{{2f_{0} }}{{NBW}}} \right\rceil$$

While $\:{f}_{0}$ is the center frequency and $\:NBW$ is the null bandwidth,$\lceil\blacksquare\rceil$provides the maximum integer greater than a given number.

Numerous prior studies on wavelet transform processors (WTPs) have employed configurations with significantly more input IDT fingers than output ones^16–19^, resulting in increased processing time and larger substrate dimensions. In contrast, the present approach adopts a balanced finger count for both input and output IDTs, as outlined below.

As reported by Brian Russell et al.^13^, the equation is given as follows:

5 $$varDelta\:{f}_{-3dB}=\frac{0.883{f}_{0}}{k}$$

Here k, the cycle index at which the power spectral density is most densely distributed. Equation (4) into (5), getting the number of input-output finger transducers. Tables 1 and 2 provide an overview of the parameters used in the simulation.

**Table 1:** Physical properties of selected materials.

Substrate material	velocity$\:{(\varvec{v}}_{\varvec{s}}\varvec{m}/\varvec{s})$	$\:{\varvec{K}}^{2}\left(\varvec{\%}\right)$	$\:{\varvec{C}}_{0}\left(\varvec{p}\varvec{f}/\varvec{c}\varvec{m}\right)$
ST-quartz	3158	0.11	0.55

Table 1 is integral to the simulation framework, presenting key physical properties of the substrates employed. It includes wave speed, capacitance per unit length (C₀), and the electromechanical coupling coefficient (K²). Notably, C₀ plays a critical role in the Mason model’s equivalent circuit, where it directly influences the computation of susceptance and conductance.

**Table 2:** Design parameters

**Frequency (**MHz**)**	**NBW** (MHz)	**N(I/O)**	**(MHz)**	**S**
1747.5	40	87	17.736	0.0075

ST-quartz was selected as the substrate material due to its favorable thermal stability and inherently low electromechanical coupling coefficient. This choice enhances the reliability of the WTPs’ communication system. The low coupling coefficient of quartz also contributes to reduced electrode reflections and minimal passband ripples, thereby improving overall filter performance.

The formulation of the wavelet envelope function at the specified scale of 0.0075 is presented below^19^:

6 $${A}_{0.0075}=11.547exp\left(-{\frac{1}{2{s}^{2}}t}^{2}\right)$$

Details of the acoustic aperture lengths for the input fingers are presented in Table 3.

**Table 3:** Design specifications for the aperture lengths(µm) for input IDTs at S = 0.0075.

No. of finger	$\:{H}_{0}$	$\:{H}_{2}$	$\:{H}_{3}$	$\:{H}_{4}$	$\:{H}_{5}$	$\:{H}_{6}$	$\:{H}_{7}$	$\:{H}_{8}$	$\:{H}_{9}$	$\:{H}_{10}$	$\:{H}_{11}$	$\:{H}_{12}$	$\:{H}_{13}$
w	400	399.85	399.41	398.68	397.66	396.36	394.77	392.92	390.80	388.43	385.82	382.97	379.90
No	$\:{H}_{14}$	$\:{H}_{15}$	$\:{H}_{16}$	$\:{H}_{17}$	$\:{H}_{18}$	$\:{H}_{19}$	$\:{H}_{20}$	$\:{H}_{21}$	$\:{H}_{22}$	$\:{H}_{23}$	$\:{H}_{24}$	$\:{H}_{25}$	$\:{H}_{26}$
w	376.62	373.14	369.49	365.67	361.70	357.59	353.35	349.02	344.59	340.09	335.53	330.92	326.28
No	$\:{H}_{27}$	$\:{H}_{28}$	$\:{H}_{29}$	$\:{H}_{30}$	$\:{H}_{31}$	$\:{H}_{32}$	$\:{H}_{33}$	$\:{H}_{34}$	$\:{H}_{35}$	$\:{H}_{36}$	$\:{H}_{37}$	$\:{H}_{38}$	$\:{H}_{39}$
w	321.63	316.98	312.34	307.73	303.15	298.63	294.16	289.76	285.45	281.22	277.09	273.05	269.13
No	$\:{H}_{40}$	$\:{H}_{41}$	$\:{H}_{42}$	$\:{H}_{43}$	$\:{H}_{44}$								
w	265.33	261.64	258.07	254.63	251.32								

### Output IDTs computation

**Fig. 1:**
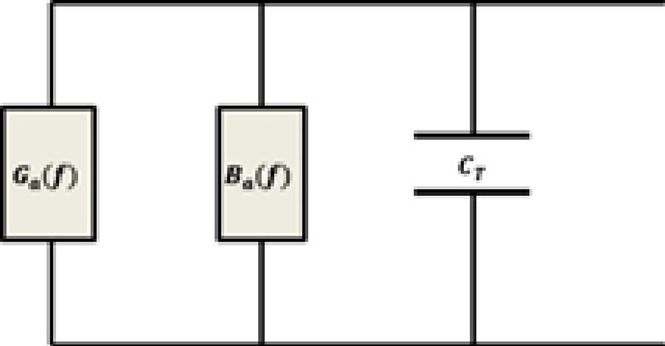
Equivalent circuit model for SAW device.

The output IDTs are conventionally structured with uniform finger overlap and spacing, consisting of 87 fingers. The computational procedure adheres directly to Mason’s model, as outlined below in Fig. 1:

Here, $\:{G}_{a}\left(f\right)$ and Ba(f) denote the radiation conductance and radiation susceptance, respectively, while $\:{C}_{T}$represents the total static capacitance. Near the center frequency, the contribution of radiation conductance is excluded from the formulation^4–6,20^.

7 $${G}_{a}\left(f\right)={G}_{a}\left({f}_{0}\right){\left(\frac{{sin}\left(X\right)}{X}\right)}^{2}$$

Where $$\:{G}_{a}\left({f}_{0}\right)=8{K}^{2}{f}_{o}{C}_{T}N$$

8 $${B}_{a}\left(f\right)={G}_{a}\left({f}_{0}\right)\frac{\text{sin}\left(2X\right)-2X}{2{X}^{2}}$$

While $\:\:{B}_{a}\left(f\right)$is the radiation susceptance.

9 $$X=N\pi\:\left(\frac{f-{f}_{0}}{{f}_{0}}\right)$$

10 $${C}_{T}={C}_{0}{W}_{a}N$$

Where $\:{W}_{a}$ is the finger overlap.

11 $${W}_{a}=\frac{4{K}^{2}N}{{R}_{in}\left(2{f}_{o}{\varvec{C}}_{0}N\right)({\left(4{K}^{2}N\right)}^{2}+{\pi\:}^{2})}$$

12 $$Y\left(f\right)={G}_{a}\left(f\right)+j\left(2\pi\:{C}_{T}+{B}_{a}\left(f\right)\right)$$

13 $$Z\left(f\right)=\frac{1}{Y\left(f\right)}$$

Equations (12) and (13) represent the admittance and impedance, respectively, of the frequency response of the output represented by

14 $$H\left(f\right)=4{K}^{2}{f}_{o}{C}_{T}Ns{inc}^{2}\left(X\right)\:\:{e}^{-i\left(\frac{N+D}{{f}_{o}}\right)}$$

**Fig. 2:**
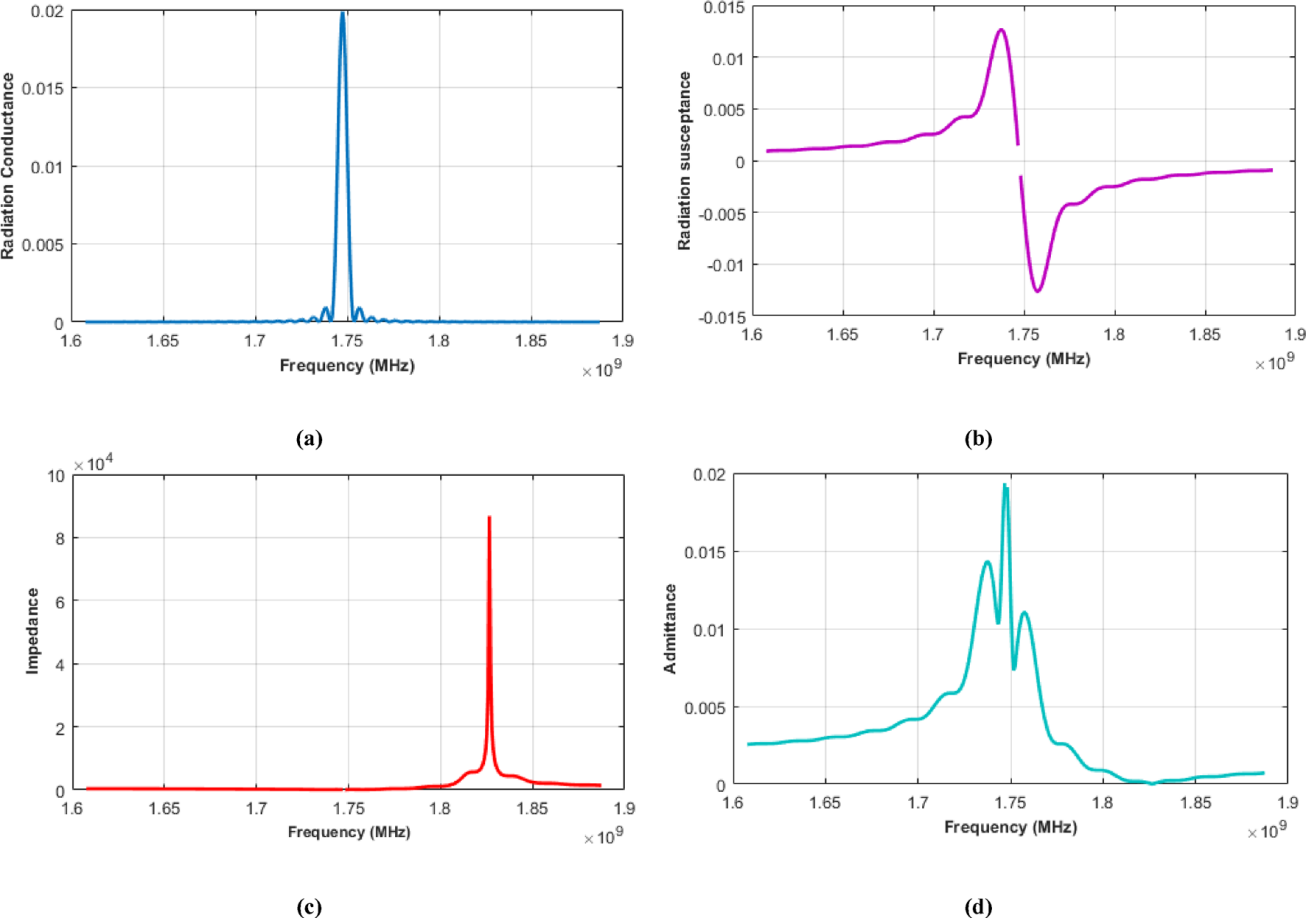
ECM parameters for output IDT(**a**) Radiation conductance. (**b**) Radiation susceptance. (**c**) Impedance. (**d**) Admittance.

The output conductance and susceptance of the filter centered at 1474.5 MHz, with 40 MHz null bandwidth derived from Eqs. (7) and (8), are illustrated in Figures 2a and b, respectively, while Figures 2c and d represent the impedance and admittance at output IDT.

**Fig. 3:**
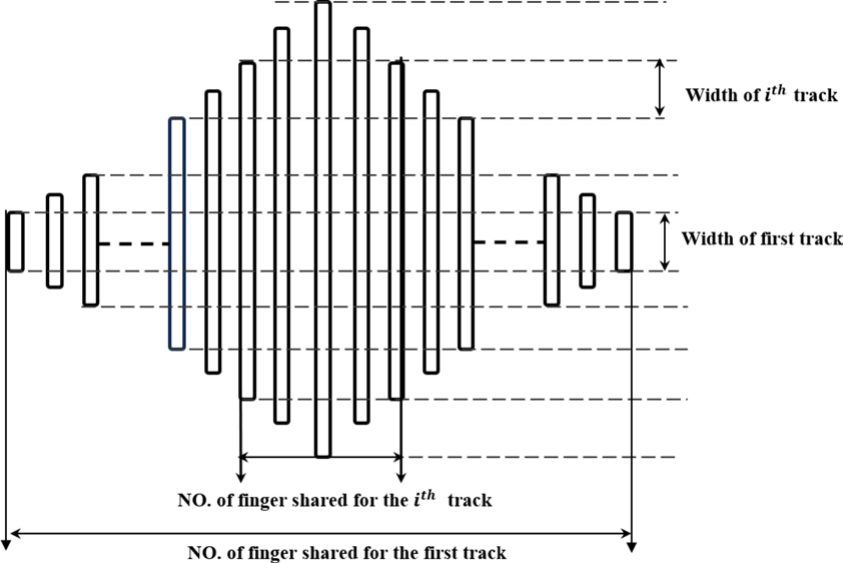
Mechanism for segmenting Input IDTs.

Since the input IDTs of the WTPs utilize apodized structures, while the output IDTs adopt regular configurations with uniform finger width and spacing, the associated parameters cannot be directly computed using the same formulas applied to the output IDTs. In accordance with^2^, the apodized IDTs are segmented into discrete tracks, as illustrated in Fig. 3. Each track is treated as an equivalent regular IDT, allowing the standard formulation for regular IDTs to be applied. The overall parameter computation is then obtained by summing the corresponding matrices across all tracks.

**Fig. 4:**
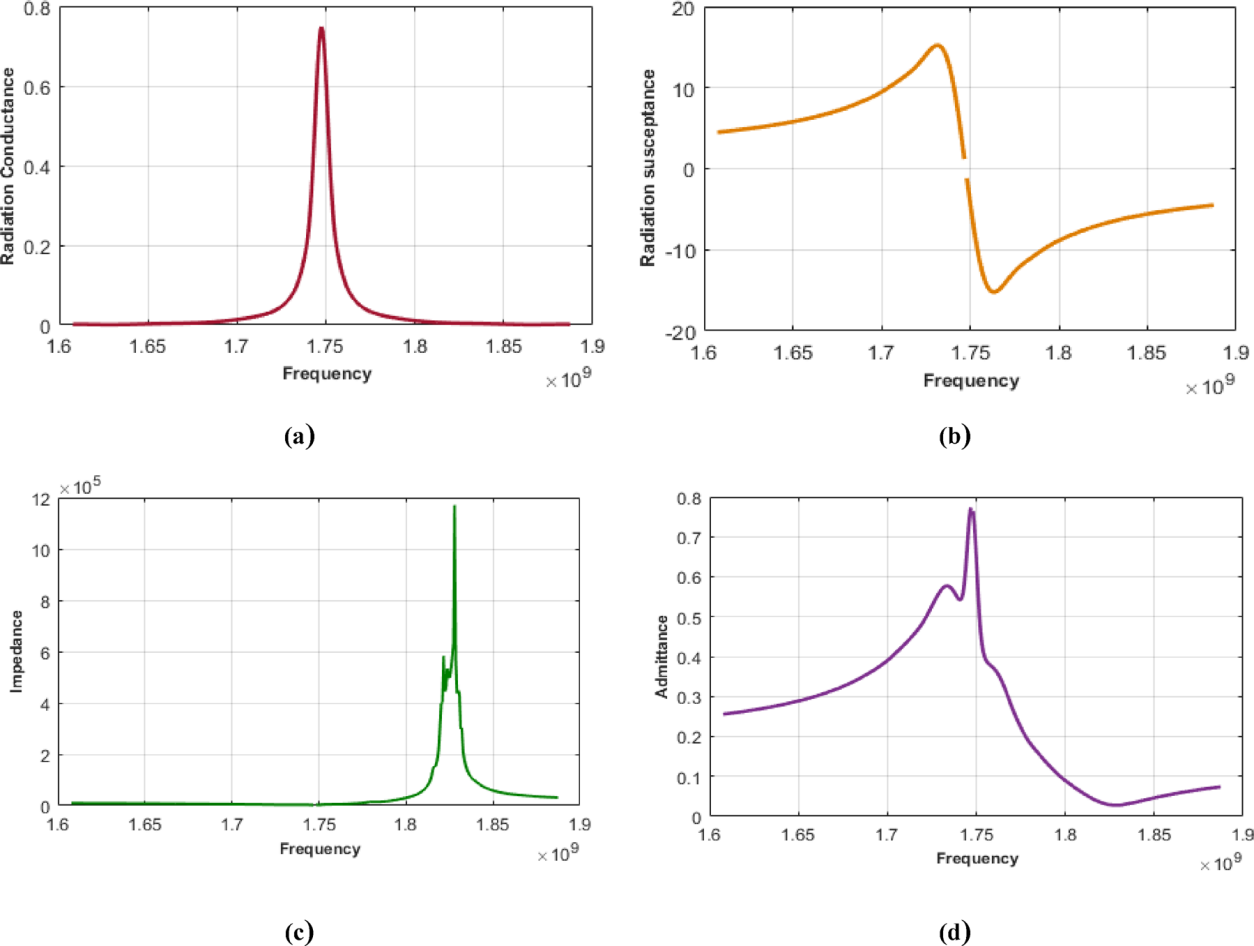
ECM parameters for input IDT(**a**) Radiation conductance. (**b**) Radiation susceptance. (**c**) Impedance. (**d**) Admittance.

As shown in Fig. 3, a defined number of fingers is shared across tracks; this method is restricted to scenarios involving an odd number of fingers. The geometric symmetry centered around the longest finger enables path division based on pairs of fingers of equal length positioned symmetrically on either side. The number of fingers contributing to a given path is determined by subtracting twice the finger’s order (minus one) from the total finger count. Subsequently, the complete matrix comprising aperture lengths and their associated shared fingers is prepared for application of the ECM equations This produces matrices corresponding to conductance, susceptance, admittance, and impedance, each structured with dimensions m×n, where m denotes the number of tracks and n corresponds to the number of simulation samples. Figure 4 shows the ECM’s calculation parameters for input IDTS.

**Fig. 5:**
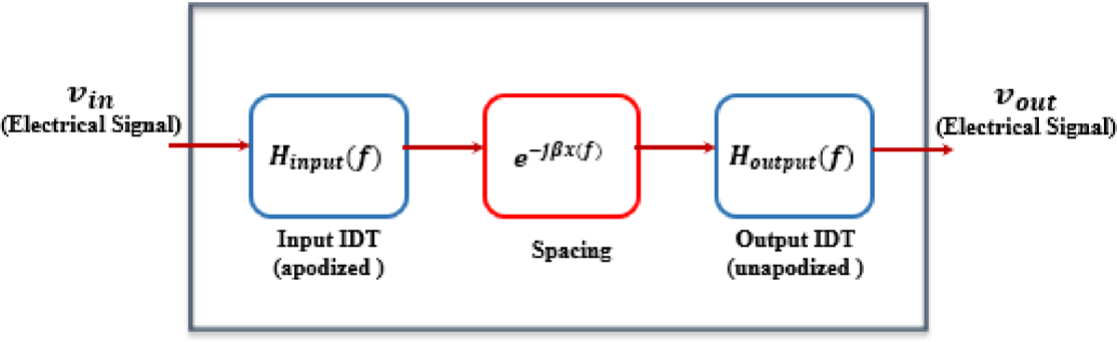
The calculation stages of a total frequency response single-stage design.

To determine the overall frequency response of a single stage depicted in Fig. 5 and defined by Eq. 15.

15 $$\left|{H}_{singl-stage}\left(f\right)\right|=\left|{H}_{I}\left(f\right)\right|\left|{H}_{O}\left(f\right)\right|$$

Here, $\:{H}_{I}\left(f\right)$ and $\:{H}_{O}\left(f\right)$ denote the frequency responses of the input and output IDTs, respectively, and are computed as described in^3–6^. The spatial interval between input and output fingers, expressed as x(f), is frequency-dependent; however, under uniform finger spacing, it may be approximated by a constant distance d, characterized as the distance between the central axes of the input and output IDTs^13^.

**Fig. 6:**
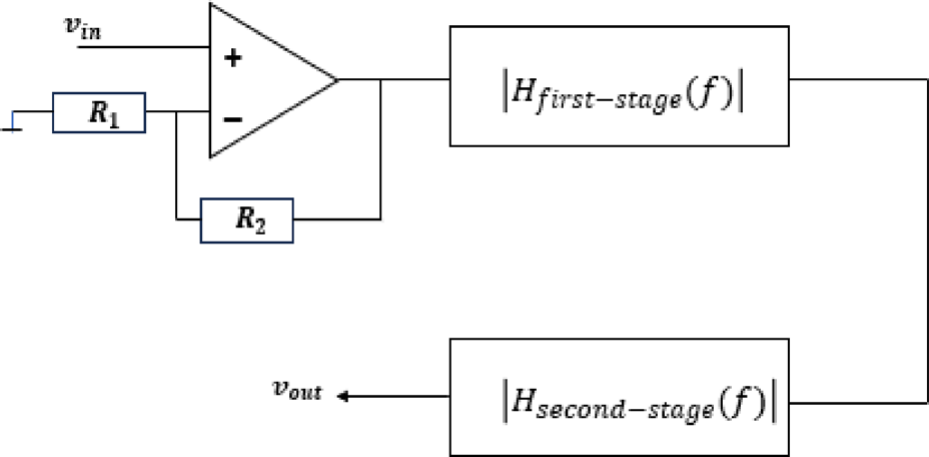
The overall stages of the filter design.

In contrast, the multi-stage design is presented in Fig. 6 alongside Eq. (16).

16 $$\left|{H}_{multi-stage}\left(f\right)\right|={\left|{H}_{first-stage}\left(f\right)\right|}^{\:}\:\left|{H}_{second-stage}\left(f\right)\right|$$

Figure 6 illustrates the two-stage filter design, where each stage comprises a balanced WT/SAW filter. To compensate for the amplitude attenuation introduced by the wavelet transforms, the input signal is amplified before filtering^18^. After amplification, the resulting output is given by$\:\frac{{\varvec{R}}_{2}}{{\varvec{R}}_{1}}{\varvec{v}}_{\varvec{i}\varvec{n}}.$.

## Comparative evaluation and the improved response characteristics

**Fig. 7:**
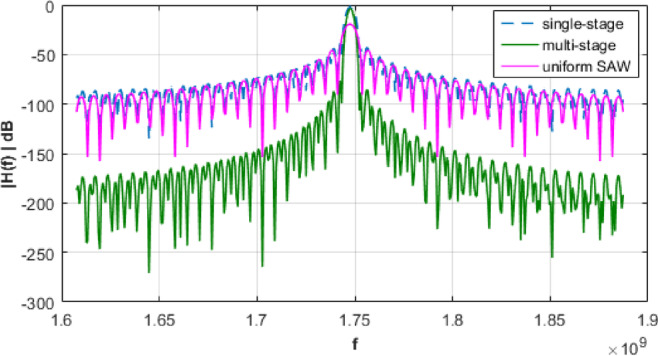
Total frequency response of filter$\:{f}_{0}=1747.5MHz$.

This section compares frequency response characteristics, explores methods to enhance the frequency response, includes a comparison with selected commercially available filters, and discusses anticipated performance discrepancies in physical implementation. Starting with a comparison that directly benchmarks the performance of the proposed method against established techniques, as illustrated in Fig. 7. The figure presents the uniform SAW design (with unapodized input and output IDTs), the single-stage Morlet apodized design, and the proposed multi-stage design.

The multi-stage design demonstrates clear superiority over the single-stage and uniform SAW techniques in both attenuation depth and spectral shaping. Including the uniform SAW design in this comparison enhances the manuscript by offering a broader context and benchmarking the proposed method against a well-established alternative. This comparative analysis highlights the effectiveness of the multi-stage approach, confirming its strong potential for advanced RF and microwave signal processing applications.

To improve the bandpass characteristics of signal processing filters, window functions play a critical role in shaping the frequency response. Ideal window functions exhibit a narrow main lobe in the frequency domain, coupled with side-lobes that decay rapidly, thereby minimizing spectral leakage and improving selectivity. A wide range of window functions is available to accommodate diverse design specifications, including the Kaiser, Hamming, Hann, and Gaussian windows. Each offers distinct trade-offs between main lobe width and side-lobe attenuation, allowing designers to tailor the filter response to specific performance criteria Table 4^21–24^.

**Fig. 8:**
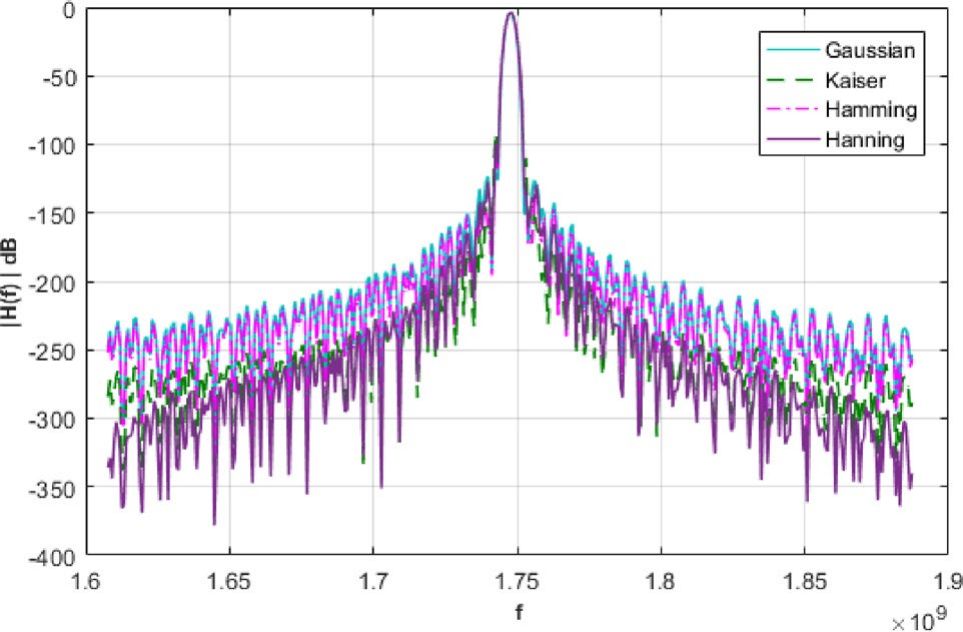
A Comparison of different window functions.

Measured insertion losses for single-stage and multi-stage systems are − 1.7456dB and − 3.4913dB, respectively. Figure 8 presents a comparative analysis of various window functions incorporated into the multi-stage filter design response.

**Table 4:** Simulation results related to different window functions.

Window function	Expression	Relative sidelobe attenuation(dB) (single-stage)	Relative sidelobe attenuation(dB) (multi-stage)
Gaussian	$\:\:\:\:\:\:w\left(n\right)={e}^{-\frac{1}{2}{\left(\frac{\alpha\:n}{(N-1)/2}\right)}^{2}}$	−78.2	−122.8
Hamming	$\:\:w\left(n\right)=0.54-0.46\text{cos}\left(2\pi\:\frac{n}{N}\right)$	−73	−136
Hanning	$\:w\left(n\right)=0.5(1-\text{cos}(2\pi\:\frac{n}{N}))$	−65.5	−132.2
Kaiser	$\:w\left(n\right)=\frac{{I}_{0}\left(\beta\:\sqrt{1-{\left(\frac{n-N/2}{N/2}\right)}^{2}}\right)}{{I}_{0}\left(\beta\:\right)}\:\:\:\:\:\:$	−58.5	−142.9

**Fig. 9:**
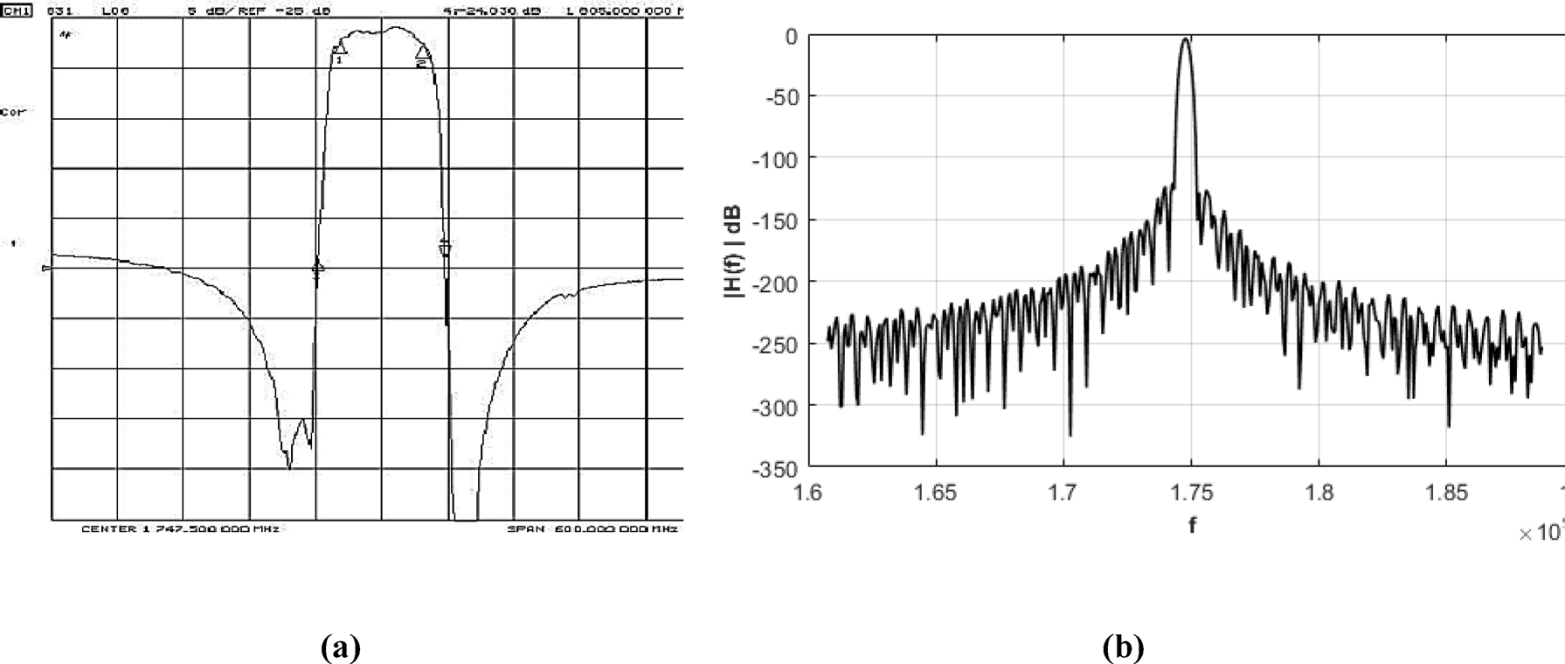
Comparison of the simulated and commercial device with the same center frequency. (**a**)Commercial device^25^(**b**) Simulated.

The results were further compared to those of a commercially available device operating at the same center frequency. To support this comparison between simulated results and measured data from real devices, references^25,26^ have been included. Evaluation of the commercial models “AM1747B1467” by Anatech Electronics Inc. (a ceramic bandpass filter) and “SF2133E” by RFMI (a SAW filter) reveals a high level of agreement when analyzed using our program framework. The results and comparisons are presented in Fig. 9, as well as in Table 5.

**Table 5:** A comparison between a commercial device and a simulated one.

Parameter	AM1747B1467	SF2133E	Simulated
$\:{f}_{0}$	1747.5 MHz	1747.5 MHz	1747.5 MHz
IL(MAX)dB	3.0	4.0	3.49
Side-lobe attenuation(dB)	50	25	122.8
Dimensions	13 × 12 mm	3 × 3 mm	0.6 × 1.0 mm

This table presents a comparative analysis of key performance parameters between two commercially available filters—AM1747B1467 (ceramic bandpass) and SF2133E (SAW)—and the simulated design. All three models share the same center frequency of 1747.5 MHz, ensuring a fair benchmark. The simulated filter demonstrates competitive insertion loss (IL) at 3.49 dB, closely matching the ceramic filter and outperforming the SAW filter. Notably, the simulated design achieves significantly superior side-lobe attenuation at 122.8 dB, compared to 50 dB and 25 dB for the ceramic and SAW filters, respectively, indicating enhanced signal selectivity. Additionally, the compact dimensions of the simulated structure (0.6 × 1.0 mm) highlight its potential for integration in space-constrained applications, offering a substantial size reduction over the commercial counterparts. Overall, the simulated design shows promising improvements in both performance and footprint.

The simulation results—especially the frequency response in Fig. 11(b) and the side-lobe attenuation values in Table 4 highlight the theoretical capabilities of the wavelet-integrated multi-stage SAW filter architecture. Nonetheless, translating this idealized performance into a physical device introduces unavoidable manufacturing and material constraints that lead to deviations from the simulated model. Such discrepancies are a recognized challenge in bridging the gap between theoretical design and practical realization in high-frequency RF systems. Certain factors influence side-lobe attenuation, while others primarily impact insertion loss. despite those, our selection of the current design reflects a deliberate effort to mitigate adverse effects and closely align the simulated performance with the practical behavior of the filter in real-world implementation. In real-world SAW devices, part of the surface acoustic wave energy is converted into bulk acoustic waves (BAWs), leading to reduced out-of-band rejection. Increasing the number of IDT finger pairs helps suppress BAW generation and mitigate its impact^3,17^. This design strategically utilizes an alternative geometric configuration with an increased number of IDT fingers to suppress bulk acoustic wave (BAW) effects and enhance side-lobe attenuation. Also, acoustic reflections at IDT edges and substrate boundaries caused by fabrication imperfections like uneven electrode thickness or material inconsistencies generate unwanted signals that elevate the noise floor and diminish stopband attenuation. The simulated insertion loss (IL) of 3.49 dB aligns well with commercial standards. In practice, IL may rise due to material-dependent electromechanical coupling coefficients (ECC) and electrode ohmic losses. Quartz, with its low ECC and zero temperature coefficient of delay (0 ppm/C°)^20^, offers excellent thermal stability and helps limit IL. However, at high frequencies, reduced electrode dimensions increase resistance, causing RF energy dissipation and further IL. While quartz ensures stable wave propagation, managing thermal effects on electrode design remains essential for optimal filter performance.

## Time domain analysis

COMSOL Multiphysics serves as an advanced simulation environment that combines finite element analysis (FEA), numerical solvers, and Multiphysics modeling capabilities, making it suitable for a broad spectrum of physics and engineering applications involving coupled phenomena. In this study, COMSOL is utilized to conduct FEM analysis of the WT/SAW filter. FEM has been used previously in many applications for signal or wave detection^27,28^. The filter geometry is modeled on a quartz-type substrate to accurately capture the piezoelectric and acoustic wave propagation characteristics. The filter’s displacement is monitored both across the thickness of the piezoelectric substrate and along the direction of wave propagation. A piezoelectric material is employed to facilitate the direct transduction between electrical and mechanical energy, leveraging the property that the free surface of an elastic medium can support the generation of surface acoustic waves. Table 6 outlines the input parameters defined for the COMSOL simulation model. According to the fundamental principles of surface acoustic wave (SAW) propagation, the behavior of SAWs within a piezoelectric substrate is governed by both stress and strain relations, resulting in a coupling between electrical and mechanical properties. This interaction can be formally represented using a piezoelectric constant matrix [e].

**Table 6:** The input parameters to the COMSOL Model.

Parameter	Expression	Value	Description
$\:{f}_{o}$	1747.5E6	1.7475 × 10^9^	Center frequency
$\:{V}_{R}$	3158[m/s]	3158	Rayleigh wave velocity
$\:\lambda\:$	$\:\frac{{V}_{R}}{{f}_{o}}$	1.8072 × 10^− 6^	wavelength
$\:{W}_{0}$	$\:\frac{\lambda\:}{4}$	4.5179 × 10^− 7^	Electrode width
gap_port	$\:5*\lambda\:$	9.0358 × 10^− 6^	Horizontal gap between ports
Pitch	λ	1.8072 × 10^− 6^	Pitch of electrodes
thick	1.5*λ	2.7107 × 10^− 6^	Substrate thickness
$\:{h}_{max}$	$\:\frac{\lambda\:}{15}$	1.2048 × 10^− 7^	Max mesh size

The finite element method (FEM) formulation is based on the constitutive Eqs. (17) and (18), as referenced in^20^.

17 $$D=\left[e\right]\left[S\right]+\left[\epsilon\:\right]E$$

18 $$\left[T\right]=\left[c\right]\left[S\right]-\left[{e}^{t}\right]E$$

In this formulation, D denotes the electric displacement vector, [c]represents the elastic stiffness matrix, [S] is the strain vector, and E corresponds to the electric field intensity. The piezoelectric coupling is characterized by a 3 × 6 matrix[e], comprising 18 components, along with its transpose $\:\left[{e}^{t}\right]$.The dielectric permittivity is described by a 3 × 3 matrix **[ε]**containing nine elements. The specific values of the piezoelectric coefficients in [e] are determined by the symmetry characteristics of the piezoelectric crystal, as outlined in the subsequent equation.

19 $$\left[e\right]=\left(\begin{array}{ccc}{e}_{11}&\:{-e}_{11}&\:0\\\:0&\:0&\:0\\\:0&\:0&\:0\end{array}\:\:\:\:\:\begin{array}{ccc}{e}_{14}&\:0&\:0\\\:0&\:{-e}_{14}&\:{-e}_{11}\\\:0&\:0&\:0\end{array}\right)$$

where $$\:{e}_{11}=-0.1710\:{e}_{14}=-0.0406$$.

The results derived from the simulation are shown in the corresponding figures.

**Fig. 10:**
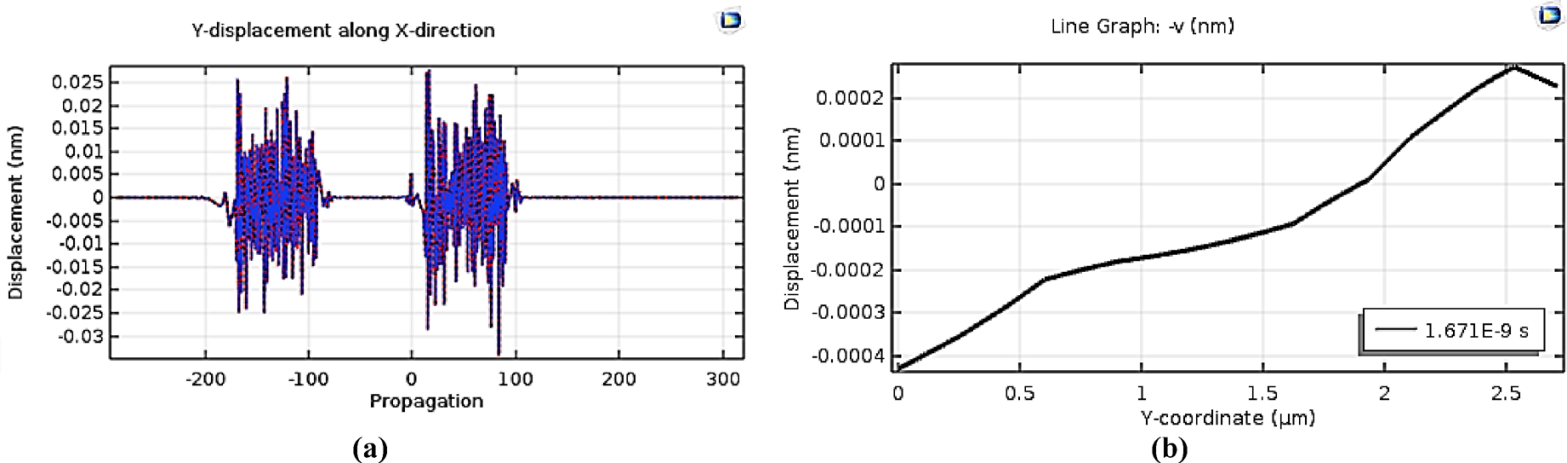
The displacement analysis (**a**) The displacement along wave direction. (**b**) The displacement along substrate thickness.

**Fig. 11:**
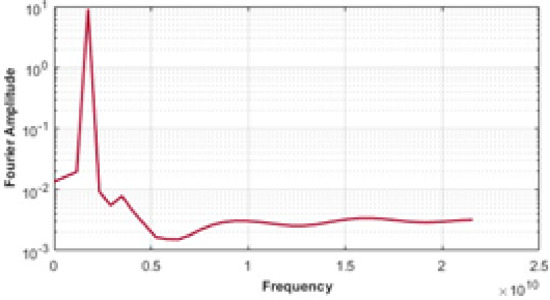
Fourier coefficient of output signal.

Figure 10 presents the displacement analysis, where Figure (10.a) shows displacement along the wave propagation direction, and Figure (10.b) depicts displacement across the substrate thickness. As illustrated in Figure (10.b), the displacement field associated with the surface acoustic wave is effectively confined within the designated piezoelectric substrate thickness of 1.5λ, exhibiting no significant penetration beyond this boundary. Furthermore, the Fourier coefficients of the output signal are evaluated in Fig. 11.

For the quartz substrate, characterized by a surface acoustic wave propagation velocity of 3158 m/s and a center frequency of 1747.5 MHz, the corresponding wavelength is calculated to be 1.8072 μm. Accordingly, the center-to-center spacing between the input and output interdigital transducers (IDTs) is determined to be 87.1948 μm. The signal processing time for a single-stage configuration is 27.61 ns. The overall substrate dimensions for the multi-stage are specified as 0.6 mm in width and 1 mm in length.

## Conclusion

This study presents a high-precision wavelet-integrated surface acoustic wave (SAW) bandpass filter centered at 1747.5 MHz, tailored for LTE uplink applications. By leveraging wavelet transform techniques in both the design and analysis phases, the proposed filter architecture achieves enhanced spectral resolution, effective side-lobe suppression, and precise null bandwidth control. The incorporation of Gaussian, Kaiser, Hamming, and Hanning window functions enhances frequency selectivity and effectively suppresses spectral leakage. Finite element modeling using COMSOL Multiphysics validates the electromechanical behavior of quartz substrates, while MATLAB-based wavelet-domain processing confirms strong frequency selectivity and low insertion loss. Comparative analysis between single-stage and multi-stage configurations demonstrates the superior performance of the latter in terms of side-lobe attenuation and passband sharpness. The filter exhibits stable center frequency alignment, robust energy confinement, and compact substrate dimensions, affirming its viability for next-generation RF front-end systems in mobile communication.

## Data Availability

All data generated or analyzed during this study are included in this published article.
